# Nothing Else Matters! Trade-Offs Between Perceived Benefits and Barriers of AAL Technology Usage

**DOI:** 10.3389/fpubh.2019.00134

**Published:** 2019-06-12

**Authors:** Julia Offermann-van Heek, Martina Ziefle

**Affiliations:** Human–Computer Interaction Center, RWTH Aachen University, Aachen, Germany

**Keywords:** technology acceptance, ambient assisted living (AAL), conjoint analysis, perceived benefits and barriers, trade-offs, care experience

## Abstract

Ambient assisted living (AAL) technologies present one approach to counter the challenges of demographic change in terms of an aging population, rising care needs, and lacking care personnel by supporting (older) people in need of care and enabling a longer and more independent staying at own home. Although the number of studies focusing on AAL users' acceptance and perception has increased in the last years, trade-off decisions—the weighing of perceived benefits and barriers of technology usage—have not been studied so far. Nevertheless, this is of high relevance: A realistic evaluation of adoption behaviors in different stakeholders (patients, care personnel) requires an understanding of exactly the weighing process of benefits against the barriers in line with the decision of the final willingness to use AAL technology. The current study applied a conjoint analysis approach and investigates people's decision behavior to use an AAL system for a family member in need of care. Study participants (*n* = 140) had to decide between realistic care scenarios consisting of different options of two benefits (increase in safety, relief of caring burden of relatives) and two barriers (access to personal data and data handling) of technology usage. Results revealed data access and privacy to be most relevant for the decision to use AAL technology at home. However, care experience essentially influenced the decision patterns. For the care experienced group, data access should be limited to most trusted persons and close relatives, rather than to medical professionals. The most important reasons to use AAL are the emotional relief and the felt safety for the person in care. For care novices, in contrast, data access should be in the exclusive responsibility of medical professionals. The reasons that militate in favor of using AAL technology are the increase in process efficiency and medical safety. The results are useful to develop user-tailored technology concepts and derive user-specific communication guidelines within and across clinical and home care contexts.

## Introduction

Almost all over the world, demographic change presents major challenges for societies and health care systems: an increased longevity, lower fertility rates, and lacking care personnel lead to aging societies facing shortages in the care sector resulting in more and more older people in need of care, who have to be supported either in their home environment or in professional care institutions (1, 2). Research, industry, and policy therefore accelerate the development of technical solutions aiming for assistance and support of older people, people in need of care, their caring relatives, or professional care personnel. By means of novel technological concepts and assisting systems, the major motivation of older people is focused: being as independent as possible and staying as long as possible within the own living environment (3).

In the field of ambient assisted living (AAL) technologies and systems, a multitude of single devices, e.g., fall detection systems (4, 5) but also smart home systems (6–8), have already been developed, which are partly in use. Those systems aim for diverse functions, such as facilitating living at home by smart home elements, enhancing safety by monitoring of medical parameters or detecting of falls, or acting as medical reminders, e.g., appointments or intake of medicine (9). In spite of the technological potential of the systems and, also, in spite of their availability on the market, they have not widely been used in real life and professional care contexts so far (10). Reasons for the hampered rollout and the prevention of a broad adoption are related to concerns about the technical complexity, economic burdens, lack of support by health insurances (11–14), but also potential fears of care persons to be controlled by supervisors and colleagues (15). Beyond these practical reasons, one major source of the reluctance lies in the missing public acceptance of the systems and a deeply grained uncertainty whether those systems might bring more positive than negative changes (16) [for an overview, see Blackman et al. (3)]. In addition, care needs at older age and health issues are perceived as private and quite intimate, which might not be adequately met by technology [e.g., references Ziefle and Schaar (17), Ziefle et al. (18), and Mynatt et al. (19)]. To pave the way for future users' acceptance and technology adoption in real life, it is necessary to understand future users' perspectives and perception of assisting technologies and systems.

Previous studies in this research field have intensively focused on future users' assessments of benefits and barriers of assisting technologies [e.g., references van Heek et al. (15), Peek et al. (20), Offermann-van Heek and Ziefle (21), and Beringer et al. (22)], consumer evaluations of specific systems and single devices [e.g., Peek et al. (20), Larizza et al. (23), and Buckley et al. (24)], and also on diverse users' interaction issues with assisting technology (25). In contrast to these studies, people's acceptance decisions after weighing of benefits and barriers of technology usage have not been investigated so far—in particular not for the perspective of using an AAL system in the home of a family member in need of care. This is of utmost importance as real decisions and adoption of technologies are usually based on an individual weighing and trade-offs between beneficial and disadvantageous factors.

The current study applied a conjoint analysis approach and aimed for analyzing people's decisions between diverse benefits and barriers of using an AAL system for a family member in need of care. An online survey was conceptualized asking people (*n* = 140) of different ages for their acceptance and decision behavior regarding the usage of an AAL system. The participants were asked to decide in different scenarios which aspects are most important for them: potential benefits of AAL technology usage (here: increase in safety and relief for caring relatives) or potential barriers (here: data access and data handling).

The paper is structured as follows: within the ongoing introduction part, an overview of previous work in the field of AAL technologies and user acceptance is given. Afterwards, the empirical approach is described, introducing conjoint analysis as applied method, the selection of relevant and integrated attributes, the design of the online survey, as well as this study's sample. Subsequently, the results are presented first for the whole sample of participants, followed by a segmentation of user profiles in line with their user profile-related decision patterns. Finally, the results are discussed, limitations of the present study are critically reflected, and necessary as well as potential future work is highlighted.

## Acceptance of AAL Technologies in (Home) Care

Within the field of AAL and smart home technologies, the number of novel and innovative developments has increased intensively. A very broad spectrum of technologies (9, 26) has been developed by research and industry and is available on the market, enabling diverse functions by specific devices or more complex holistic systems. Basically, these technologies aim for support of healthy people and people in need of care [e.g., Rashidi and Mihailidis (9)]: For healthy people, AAL and smart home technologies can be used to improve and support a healthy life style or to enhance well-being. For older people and people in need of care, those technologies—meeting the care gap—are used to retain independence, to stay active, to enhance safety, and to support aging within the own home environment. Most of the technologies fulfill predominantly either medical and safety-related, automated, or communicative functions, while holistic systems aim for covering preferably all of these functional areas. Within medical functions, the focus lies clearly on monitoring and fall detection systems enabling personal alarms or emergency calls by using video- or sensor-based technologies and systems [e.g., Stone and Skubic (27), and Cheng et al. (28)]. A further aim is a barrier-free and independent communication with friends, family, or caregivers enabled by telemedicine, telecare, or videoconferencing services and technologies (29). In the field of home automation, smart home applications but also automated memory aids [e.g., Hristova et al. (30)], e.g., for intake of medicine, appointments, or remembering of daily routines [e.g., Hossain et al. (31)], are promising applications.

Along with the technical development, the awareness and relevance of users' acceptance of supporting AAL solutions and the need of user-centered technology development have also increased, indicated by rising numbers of studies referring to perception and acceptance of those technologies (20). Thereby, investigating different user groups' acceptance and intention to use diverse assisting technologies as well as understanding of users' evaluations of technology-related benefits and barriers have been focused. Existing research studies cover a broad range of user perspectives, e.g., considering older mainly “healthy” people [e.g., Beringer et al. (22), Demiris et al. (32)], older people in need of care (33), e.g., suffering from dementia (25), or professional (15, 23) as well as family caregivers (24, 34). In the majority of the studies, the participants evaluated different types of benefits, but also their concerns allowing statements and conclusions about the participants' agreement or rejection of each single aspect. With regard to perceived benefits, feeling of increased safety, increased autonomy, and a longer staying at home for older people and people in need of care represented the most relevant motives to use AAL and smart home technology [e.g., van Heek et al. (15), Peek et al. (20), and Beringer et al. (22)]. While these results were valid independent from different user groups, the perception of barriers differed for diverse user groups: For older adults, dependency on technology and a lack of personal contact represented central concerns [e.g., Beringer et al. (22), and Demiris et al. (32)]. Family caregivers, in contrast, were concerned about maintaining security at home (24), while professional caregivers identified data security, perceived control, and the invasion of privacy as major hurdles (15, 23). For professional caregivers, the acceptance of AAL technology strongly depended on the care area with different perceptions between geriatric care, nursing care, and care of people with disabilities (21).

Research has also shown that acceptance and perception of assisting technology are impacted by user diversity: Gender, age, previous experience with care, or attitude toward technology influence the acceptance of AAL technology. In the context of an aging population, age and attitudes toward aging have been shown to be relevant (35, 36). Further, research revealed significant effects of age and health status on diverse technologies (37, 38). In more detail, older adults showed higher concerns regarding potential barriers of using assisting technologies compared to younger people, while the perception and evaluation of benefits did not differ significantly (39). In the context of data privacy, a recent study (40) reported that older users were more willing to share medical data on general health and physical illnesses in comparison to younger users, given that the data are used for a global societal benefit. In addition, research indicated that also expertise with health and care influences the perception of assisting technology (41).

Beyond these insights, the trade-offs between potential benefits and barriers of AAL technology usage for people in need of care have not been investigated so far. The determination of trade-offs—thus the understanding which of the benefits and which of the barriers form the final acceptance decision to which extent—is though essential. In real-life decisions, this exactly happens. Persons might accept some barriers under certain usage conditions (e.g., sharing personal data in order to have a high safety for the person in care). Likewise, some barriers could be so elementary that they are weighed stronger than any benefits under certain circumstances or in specific user groups. Thus, when it comes to the decision to accept AAL technology in homecare, we need an understanding about people's decision behavior—when benefit- and barrier-related factors have to be weighed against each other directly (1). This indicates the necessity of an empirical approach focusing on an investigation of people's decisions behavior when using AAL technology for a person in need of care. Furthermore, it should be studied whether decisions are influenced by user diversity (3). The current study addresses those two research gaps by applying a conjoint analysis approach, which is introduced in detail within the next section.

## Objectives and Aim of the Study

The present study aimed for a holistic investigation of laypeople's decisions between selected perceived benefits and barriers of AAL technology usage taking the perspective of a caring relative into account. Further, it is investigated if user profiles can be identified differing in their underlying decision patterns.

The study was planned as an exploratory study: As the current knowledge regarding trade-offs between benefits and barriers and the relative weight and interdependence of relevant factors was not yet prevailing, no specific hypotheses could be formulated. Therefore, the study focused on an investigation of relevant factors, their relationships, and their impact on acceptance decisions. The underlying research questions are the following:

RQ1: Which benefit- or barrier-related criterion is most decisive: increase in safety, relief of relatives, data access, or data handling?
RQ2: Which facets of the criteria (attribute levels) contribute positively or negatively to the decisions?
RQ3: Do user profiles with different decision patterns exist?
RQ4: Which benefit- or barrier-related criterion is most decisive for whom?
RQ5: Are there any differences in the decision patterns with regard to positive or negative contributions to decisions (attribute levels)?

## Empirical Approach

This section presents the empirical approach of the current study starting with an introduction of the applied methodology of conjoint analysis. Further, the selection of relevant factors for the conjoint analysis and its empirical design are described. After introducing the conceptualization of the online survey, data analysis and the characteristics of this study's sample are detailed.

### Conjoint Analysis

The analysis and measurement method conjoint, developed by Luce and Tukey, was primarily used to design and evaluate innovative product configurations, and to identify adequate product prices and price levels (42). Beyond its original application in market research, conjoint analysis has been increasingly used for technology acceptance research, adoption behaviors, and consumers' decisions in different thematic areas since the last years, such as e.g., transportation, energy, and health care (43). Uniting a measurement model and statistical estimation algorithms, conjoint analysis allows holistic investigations of complex decisions, which depend on several relevant factors, exceeding the opportunities of conventional survey-based approaches aiming for evaluations of isolated factors. By participants' assessments of diverse scenarios, more realistic decision situations can be simulated, in which several factors are weighed against each other. Methodologically, the scenario configurations consist of multiple attributes (e.g., product price) and differ from each other in their attribute levels (e.g., “$100” vs. “$120” vs. “$140”) (44). Based on the identification of a relevant set of attributes and their attribute levels, decision processes are simulated. Participants' decisions are subsequently decomposed into importance (weight of attributes) and part-worth utilities of the attributes and their levels (45, 46), indicating which of the levels contribute to which extent to the overall decision. For this study, we used the choice-based-conjoint analysis (CBC) as it aims for the imitation of complex decision processes, in which several attributes influence the final decision (44).

### Selecting Relevant Factors of AAL Technology Acceptance

The identification, selection, and definition of relevant attributes—to be evaluated in the choice tasks—are major important steps during the conceptualization, preparation, and implementation of a conjoint study (47, 48). A limited complexity is necessary as it impacts the generalizability and significance of findings, and in order not to overstrain participants.

For the present study, relevant benefit- and barrier-related factors were selected on the basis of a literature analysis and preceding studies in the specific research field of AAL acceptance, which identified and analyzed perceived barriers and risks regarding usage of AAL technologies in private home, [e.g., Peek et al. (20)] as well as professional care environments [e.g., Offermann-van Heek and Ziefle (21)]. In the following, we describe how the barriers and the benefits of using AAL technology at home were selected (for an overview, all attributes and their levels are detailed in Table 1).

**Table 1:** Attributes and levels used in the conjoint study.

**Attribute**	**Number of levels**	**Attribute levels**			
Data access	4	Trusted persons	Relatives	Medical experts	Emergency services
Data handling	4	Real time (no storage)	Short term (1 week)	Middle term (1 month)	Long term (permanent)
Safety	4	Fast	Medical	Structuring	Felt
Relief of caring relatives	4	Temporal	Organizational	Financial	Emotional

#### Selection of Barriers

Within potential barriers and considerations of risks, the *handling of processed or recorded data* represents an essential topic. As data security-relevant aspect, the duration of data storage is frequently mentioned and critically discussed by diverse user groups (15, 49). Thus, the duration of data storage represents an attribute that was integrated as potential barrier-related factor for AAL technology acceptance within the applied conjoint analysis approach. Data handling was conceptualized using four attribute levels representing different gradations of data handling periods referring to the AAL system's usage:

1. a **real time processing** of data (data are not stored)
2. a **short-term storage** (up to 1 week)
3. a **middle-term storage** (up to 1 month)
4. a **long-term storage** of data (permanent)

As important information, different gradations of data handling periods are related with different opportunities to monitor health status and care needs of a person. For example, a long-term storage of data allows detailed analyses of health/disease progressions or analyses of movements and behavior (in particular important for patients who suffer from dementia or Alzheimer's disease). In contrast, a real-time processing of data only allows emergency calling or situational analyses. Descriptions and explanations of these interrelations were—of course—part of a detailed introduction the participants received prior to the conjoint decision tasks.

Another privacy-related barrier—being relevant for AAL technology acceptance and usage—refers to *access of data for third persons*. In this regard, several studies revealed that concerns about data access and a perceived limited individual privacy influenced the acceptance and evaluation of assisting technology negatively [e.g., Wilkowska et al. (50) and Steggell et al. (51)]. One of the most central questions in handling of health data referred to the aspect with whom data concerning the usage of an AAL system are shared (52). Hence, data access represents a second privacy- and data security-relevant aspect and was integrated as a barrier-related attribute in the conjoint study design. For the graduation of data access levels, persons with a different proximity to the person using an AAL system were chosen:

1. **trusted persons** (who are defined and selected by the person who is using the AAL system)
2. **a circle of relatives** (consisting of several people)
3. **medical experts** (such as the family doctor)
4. **emergency services** (exclusively)

#### Selection of Benefits

Considering benefits of using an AAL system, one of the most frequently mentioned aspects relates to *increased security*. Previous research in the field has proven “increase in safety” as a relevant benefit and an influencing factor for the acceptance of assisting technology [e.g., Peek et al. (20)]. In more detail, previous studies revealed that participants showed a higher willingness to accept privacy restrictions when using AAL technology provided increased safety [e.g., Wild et al. (53)]. Based on this knowledge, increase in safety was integrated as a first benefit-related attribute in the conjoint analysis approach. Within the studies identifying safety as a relevant benefit of AAL technology usage, diverse facets of safety have been proven to play a role for AAL technology usage. Hence, the following graduation of attribute levels were chosen:

1. **fast safety** related to fast assistance in emergencies
2. **medical safety** provided by monitoring of medical parameters
3. **structuring safety** by reminding a person (e.g., for intake of medicine or appointments)
4. **felt** or perceived **safety** related to the feeling that the person in need of care is not alone

Another frequently discussed benefit of using AAL technology is as follows: the relief of caregivers (family members as well as care personnel) by using AAL technology has been identified in several studies. Previous work [e.g., Lorenzen-Huber et al. (54)] found that participants were more willing to use AAL technologies in their everyday life if technology enabled a relief of caregivers. In line with the attribute safety, diverse facets of relief were identified to be relevant and were part of the conjoint study design:

1. **temporal relief** due to time savings, meaning that it is not necessary anymore to visit the person in need of care several times a day, as once a day is sufficient
2. **organizational relief** due to minor efforts in organization and infrastructure of everyday life—combining the caring person's working life and own family with caring for a family member
3. **financial relief** by saving costs of care services or costs of drives
4. **emotional relief** by the knowledge that the system monitors the health state and contacts the caring relatives in emergencies (“there is someone watching for the person in need of care”)

### Experimental Conjoint Analysis Design

**Figure 1:**
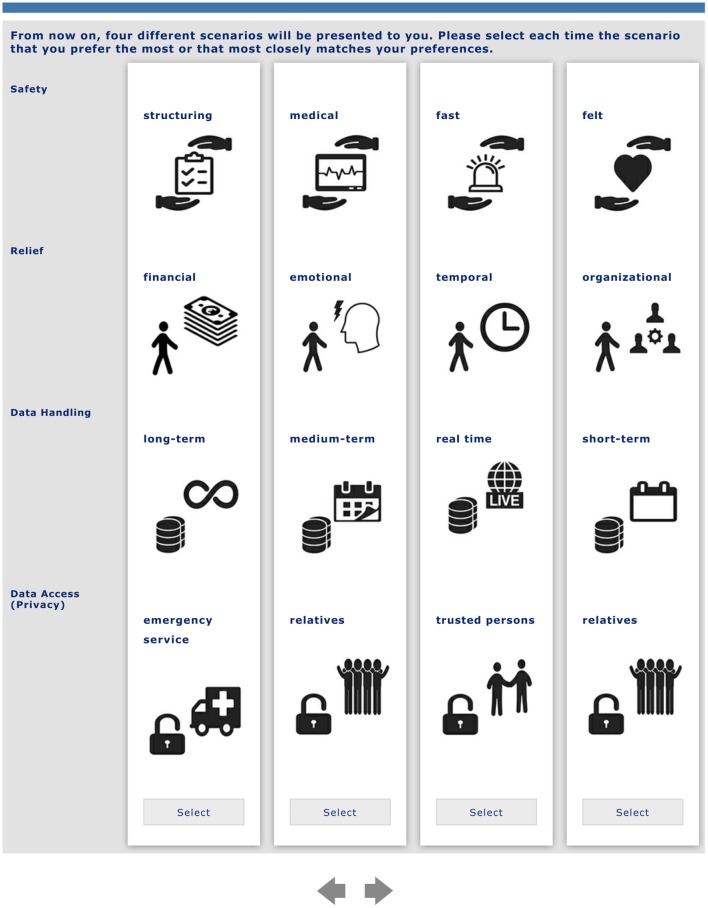
Example of a randomized decision task (translated in English, the original version was in German, the native language of participants).

As described in the previous section, four attributes with each four attribute levels were chosen to be relevant for the conjoint analysis design. Within the scenario decisions (choice tasks), four different scenario configurations were presented to the participants at a time. An example of a choice task is shown in Figure 1. In order to avoid evasive responses, a “none option” was not provided to the participants (forced-choice format). Before starting the study, each attribute (level) was explained in detail, both verbally and by pictograms, to support an easy understanding. To ensure comprehensibility the whole survey and all elements of the conjoint analysis were pretested by six participants (younger and older people) as well as checked for intelligibility and clearness. Also, the cognitive burden and the tolerable amount of decisions were tested: As a complete orthogonal design would have required all possible combinations of attribute levels (4 × 4 × 4 × 4 = 256), the conjoint analysis software provides the opportunity of reducing the choice tasks to be completed by using completely randomized choice tasks for each participant. In line with the pretests, 10 choice tasks were a reasonable number of decisions with still a satisfactory efficiency: The test of the design's efficiency yielded a median efficiency of 99% and a standard error below 05, confirming that the current design (10 randomized choice tasks per participant) was comparable to the hypothetical orthogonal design (44).

### Design of Online Survey

Before the participants started the survey, they were informed that it is of high importance for us to understand free opinions and attitudes on using AAL technology and that we were very happy and grateful if they would share their opinions with us. In addition, we stressed that (1) they are free in starting and completing the survey or not, (2) their participation was completely voluntary, and (3) monetary incentives were not offered for participation. Further, we assured a high standard privacy protection in handling the participants' data and let the participants know that data were analyzed anonymously, i.e., none of their answers can be referred to them as persons. We also explained that the submission of demographic data was completely voluntarily, and we informed all participants that their data would be deleted from our encrypted hard drives on request. Subsequent to these explanations, the participants reported to feel well informed about the study's purpose and aim as well as the freedom to quit their participation at all times. The participants confirmed that they understand the application of high privacy standards and accepted participation deliberately. As participants' privacy is a key value that our university has committed itself to uphold, this procedure was mandatory.

After starting the online survey using a link, the participants were welcomed and shortly introduced into the topic of AAL technologies. The first part of the survey asked for demographic information (age, gender, educational level, current occupation) as well as health- and care-related aspects. Regarding care experience, the participants indicated if they have passive or active experience in care by answering if they have a person in need of care in their family environment or if they have already been the caregiver for a family member in need of care (answer options: yes/no).

The second part addressed perceived benefits and barriers in a caring scenario: Enabling the assessment of the participants' perception and acceptance of using an AAL system for a family member in need of care, a detailed scenario was used. The participants were asked to imagine that one of their family members strongly depends on care and that they are the caregiver looking several times a day for this person. First, the scenario described a daily routine of the person in need of care and the family caregiver including all related duties and responsibilities of the caregiver. In a next step, it was illustrated how an implemented AAL system could support the caregiver as well as the person in need of care in their everyday life. Some exemplary functions related to reminding for intake of medicine or appointments, smart home elements, detection of falls and automatic notifications (family caregiver or—if needed—emergency services), or medical monitoring of vital parameters.

Subsequent to the scenario, the participants were asked to assess six potential benefits (α = 0.859) as well as six potential barriers (α = 0.860) of the AAL system's usage. Further, they were asked to evaluate their general perception (four items: α = 0.917) and their intention to use such an AAL system (acceptance) (three items: α = 0.738). The items (Table 2) were used to calculate overall scores for benefits, barriers, general acceptance, and intention to use (higher value = more positive evaluation: agreement).

**Table 2:** Items used to measure evaluations of benefits, barriers, general perception, and acceptance of the described AAL system (scale from “1 = I do not agree at all” to “6 = I fully agree”).

**Dimension**	**Item**
Benefit (α = 0.859)	Increased independency Longer staying at own home Facilitation of everyday life Increased feeling of safety Relief of caring relatives Fast help in emergencies
Barriers (α = 0.860)	Invasion in privacy Forwarding data to third parties Dependency on technology Recording and storage of data Replacing human care by technology Feeling of surveillance
General perception (α = 0.917)	I find the AAL system valuable I find the AAL system useful I find the AAL system beneficial I find the AAL system risky (recoded)
Acceptance (α = 0.738)	I can imagine to use the AAL system at home I like to use this AAL system I cannot imagine using such a system (recoded)

In the last part of the questionnaire, the choice tasks were applied (conjoint approach). It started with an introduction of all attributes and attribute levels of the conjoint analysis. Within this introducing part, the attributes and all their levels were briefly explained to the participants including their visualization used in the survey. The participants were asked to think about the scenario again and to empathize with the situation to be the caregiver of a family member in need of care. With regard to the choice tasks (for an example, see Figure 1), they were then asked to choose the scenario they prefer most or feel most comfortable with. In more detail, the participants each received 10 different choice tasks each consisting of four different randomized scenario configurations. For each of the 10 tasks, the participants then had to choose the scenario they liked the most in comparison to the other three presented scenario configurations.

At the end of the survey, the participants were asked to give feedback or leave comments referring to the topic and the online survey on an optional basis. The high number of comments in the open question fields showed that the participants were interested in the topic of AAL and in looking at the results, which we assured them to receive.

### Data Preparation and Analysis

In addition to descriptive (and inference statistical) analyses referring to the survey's items, the calculations of the conjoint analysis were conducted using Sawtooth Software. Based on Hierarchical Bayes analysis, the relative importance scores of attributes for the scenario decisions as well as the part-worth utilities of all single attribute levels were calculated (44). In more detail, the relative importance of an attribute indicates the extent of how important this attribute is for the scenario decision compared to the other integrated attributes. A part-worth utility value of an attribute level delivers the information about how and to what extent this level—in tendency—contributed to the scenario decisions (negatively or positively). In this regard, it should be considered that part-worth utilities cannot be compared between different attributes, but, instead, zero-centered part-worth utilities can be used to compare differences between attribute levels (45). The root likelihood (RLH) indicates the goodness of a Hierarchical Bayes model and varies between 1.0 as the best possible value and the probability of different choices in the choice tasks as the minimal score, here 0.25. For the current study, the RLH showed a sufficient goodness of fit (0.5). As a further type of analysis, Latent Class Analysis (LCA) was carried out in order to identify user segments with similar decision patterns (55, 56). More detailed information referring to this analysis can be found in the respective Results section (see the section Investigating Distinct User Groups).

### Data Collection and Characteristics of Participants

Data were collected online in Germany in June 2018. A total of *N* = 228 respondents participated in the online survey. Participants were recruited by personal contact and requests in social networks and online forums. On average, the completion of the questionnaire took 20 min. Incomplete data sets as well as data sets with implausible answering patterns were excluded from further statistical analyses. Hence, *N* = 140 were used for deeper statistical analyses.

The participants were, on average, 35.4 years (*min* = 17; *max* = 86; *SD* = 16.8) with 56.4% females (*n* = 79) and 42.9% males (*n* = 60; one person did not want to indicate gender). Asked for the participants' education level, 45.7% reported to hold a qualification for university entrance, 38.6% a university degree, and 15.7% a secondary school certificate. With regard to their current occupation, 54.3% (*n* = 76) indicated to be still in higher education, 35.0% (*n* = 49) to be employed, and 10.7% (*n* = 15) to be retired. With regard to experience in care, 36.4% reported to have a person in need of care in their closer family circle. Those people were defined to have “passive experience in care” as they witness the care of their family member. Further, 18.6% of the participants indicated to have already been the caregiver for a family member in need of care. Hence, these participants were defined to have “active experience in care.”

To understand the participants' general attitudes toward the described AAL system, evaluation scores were calculated. For this, the benefits (six items), the barriers (six items), the general perceptions (four items), and the acceptance (three items) were aggregated to a mean score, each. On average (min = 1; max = 6), the sample confirmed the benefits to use the AAL system (*M* = 4.8; *SD* = 0.7). In contrast, the barriers of the AAL system's usage were evaluated quite neutrally (*M* = 3.7; *SD* = 1.1). The participants' general perception was, on average, rather positive (*M* = 4.3; *SD* = 0.8) similar to the acceptance of the AAL system (*M* = 4.2; *SD* = 0.9).

### Results

This section starts with the decision patterns of the whole sample (relative importance of attributes and part-worth utility values for all attribute levels). Afterwards, the results of a segmentation of user profiles are presented by introducing the respective groups and their characteristics as well as their specific decision patterns.

### Decision Patterns Regarding Attributes and Their Levels

**Figure 2:**
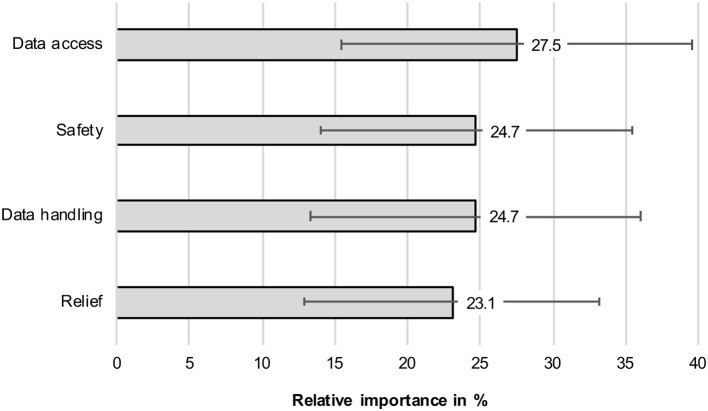
Relative importance of attributes investigating the whole sample (whiskers indicate standard deviations).

First, the relative importance of all attributes for the scenario decisions referring to the whole sample are reported (Figure 2). The relative importance indicates the relative proportion an attribute contributed to the participants' scenario decisions.

Answering RQ1, privacy operationalized as *data access* represented the most important criterion for the decisions between perceived benefits and barriers of the AAL system's usage (27.5%, SD = 12.1). The perceived benefit *safety* (24.7%, SD = 10.7) and the perceived barrier *data storage* (24.7%, SD = 11.4) were of “second” importance. In comparison, the perceived benefit *relief* (23.1%, SD = 10.2) was least important for the participants' decisions; however, the differences between the relative importance of the attributes were rather small.

**Figure 3:**
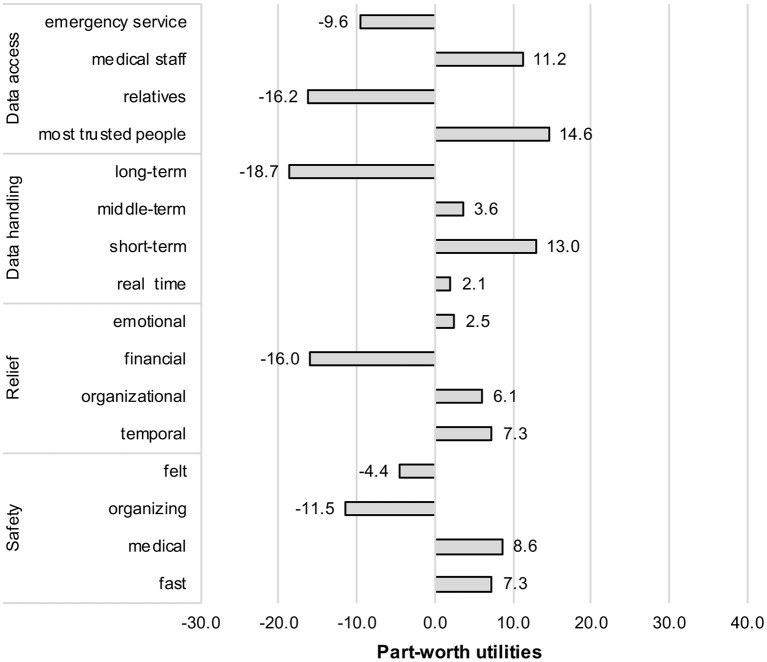
Part-worth utilities of all attribute levels.

Next, part worth utilities are reported—thus, the decision patterns within each attribute (see Figure 3). The utility values of attribute levels thereby indicate to what extent and in which direction (positively or negatively) a level contributed to the decision for a scenario, allowing it to differentiate whether the tendency between of the participants was for more or less desired alternatives (RQ2).

Starting with the evaluations within the *data access* (*privacy*) attribute, a diverging evaluation pattern emerged: data access for a defined circle of most trusted people (+14.6) received the highest utility value and was thus—compared to the other data access opportunities—most desired by the participants. Data access for medical staff (e.g., the family doctor) (+11.2) was also perceived positively. However, there were also clear-cut negative evaluations: Emergency services (−9.6) and even more clearly a broader circle of relatives (−16.2) were rejected as data receivers.

Concerning the perceived barrier *data storage*, again, a clear-cut pattern appeared: short-term storage of data (up to 1 week) (+13.0) received the highest positive utility value, whereas long-term storage (−18.7) represented unequivocally the most negative alternative. Middle-term (+3.6) data storage as well as real-time processing of data (no storage) (+2.1) received both only slight positive utility values and were therefore of minor relevance for the scenario decisions.

Considering the perceived benefit *relief of relatives* by using AAL technology, the facets temporal (+7.3), organizational (+6.1), and emotional (+2.5) relief contributed all slightly positively to the scenario decisions. In contrast, receiving (only) financial relief (−18.7) by means of AAL technology for a person in care contributed negatively to the scenario decisions and, thus, it represented the most negative alternative for the participants.

When it comes to the increase in *safety* by using AAL technology for care at home, fast safety (in terms of a fast detection of emergencies) (+7.3) and medical safety (monitoring of vital data) (+8.6) received both positive utility values and therefore made a positive contribution to the scenario decisions. In contrast, any increase of perceived safety (−4.4) was seen slightly negative. The same applied for an increased organizing safety (−11.5), which was rejected as a decisive reason to use AAL technology at home for persons in care. Apparently, the use of AAL technology should provide an increase in objective safety (detection of emergency or a higher monitoring quality in data monitoring); the mere organizational safety or the feeling of being safer does not represent reasons that militate in favor of using AAL technology at home.

So far, we portrayed the decision behavior of the whole sample without a consideration of the diversity of decisions. However, the high standard deviations with regard to the attributes' relative importance and the levels' part-worth utilities suggest an impact of different perspectives and decision behaviors.

### Investigating Distinct User Groups

As we assumed that the decision patterns might not be the same for all participants, we used LCA (55) to form user groups on the base of the scenario decisions. In contrast to a top-down analysis with predefined user segments (e.g., young vs. old participants), this bottom-up, data-driven analysis method was used in order to identify profiles of users having similar decision patterns. In addition, this type of analysis enables to identify and investigate user profiles being characterized by several user diversity factors. Hence, this allows insights in the interplay of different connected user diversity factors (such as age, living circumstances, life, or care experience) and their impact on decision behavior.

Applying the LCA procedure, the study's sample was *post hoc* divided into user groups based on similar preferences in the scenario decisions (56). The segmentation of groups includes an estimation of utilities for each group as well as the calculation of the probability each respondent belongs to the respective user group. In subsequent analyses, the identified user groups are portrayed alongside their demographic characteristics in order to derive user profiles (RQ3). A two-group segmentation showed the best data fit [criteria percentage certainty, consistent Akaike information criterion (CAIC), and relative chi square]. In the following, the user group-specific analyses are detailed starting with a description of identified user groups and their characteristics. Next, the user group-specific decision patterns and scenario evaluations are presented.

#### Characteristics of User Groups

Inference statistical analyses revealed two distinct groups with respect to diverse decision behaviors. Group 1 was significantly younger than group 2, was characterized by higher proportions of high educated participants, and consisted mainly of young academics in education. The group had a significant lower experience in care: In comparison to group 2, lower proportions of them indicated to have family members who are in need of care (passive) and have already been the caregiver for a family member in need of care (active). Summarizing, this group can be characterized as young adults who are at the beginning of their working life and have not been confronted with care responsibilities yet. This group is simply called “care novices” in the following. Table 3 shows the characteristics of the segmented user groups referring to demographic information as well as individual care experience.

**Table 3:** Characteristics of the segmented user groups.

**Variable**	**Group 1 “care novices” N = 62**	**Group 2 “care experienced” N = 78**	**Test statistics**	**Level of significance**
**DEMOGRAPHICS**				
Age [M, (SD)]	30.13 (13.71)	39.63 (17.97)	*F*_(1, 139)_ = 11.841	*p* < 0.01
Gender	58.1% female	55.1% female	*F*_(1, 139)_ = 0.023	n.s.
	40.3% male	44.9% male		
Education	Low 3.2%	Low 25.6%	*F*_(1, 139)_ = 10.286	*p* < 0.01
	Middle 50.0%	Middle 42.3%		
	High 46.8%	High 32.1%		
Occupation	Student 67.7%	Student 43.7%	*F*_(1, 139)_ = 10.286	*p* < 0.01
	Employed 29.0%	Employed 39.8%		
	Pensioner 3.2%	Pensioner 16.7%		
**CARE EXPERIENCE YES (NO)**				
Passive	27.4% (72.6%)	43.6% (56.4%)	*F*_(1, 139)_ = 3.955	*p* < 0.05
Active	11.3% (88.7%)	24.4% (75.6%)	*F*_(1, 139)_ = 3.956	*p* < 0.05

Group 2 was, on average, almost 10 years older than group 1, was characterized by higher proportions of people with lower to medium education levels, and contained significantly higher proportions of employed persons as well as pensioners. Considering experience with care, group 2 had significantly more passive but also active experience, meaning that nearly half of them have experience with a family member in need of care (passive experience). Almost a quarter have already served as caregivers for a family member in need of care (active experience). Summarizing, group 2 is more senior in care issues, has more experience in life, is still part of an active working force, or is in part already in retirement. In the following, this group is therefore simply called “care experienced.”

With respect to evaluations of benefits, barriers, general perception, and acceptance of the described AAL system, it was found that both groups differ significantly neither in their evaluations of benefits [*F*_(1, 139)_ = 0.108; *p* = 0.743, n.s.] and barriers [*F*_(1, 139)_ = 0.130; *p* = 0.719; n.s.] nor in their general perception [*F*_(1, 139)_ = 0.291; *p* = 0.590; n.s.] and acceptance [*F*_(1, 139)_ = 0.291; *p* = 0.590; n.s.] of the AAL system.

#### User Group-Specific Decision Patterns

**Figure 4:**
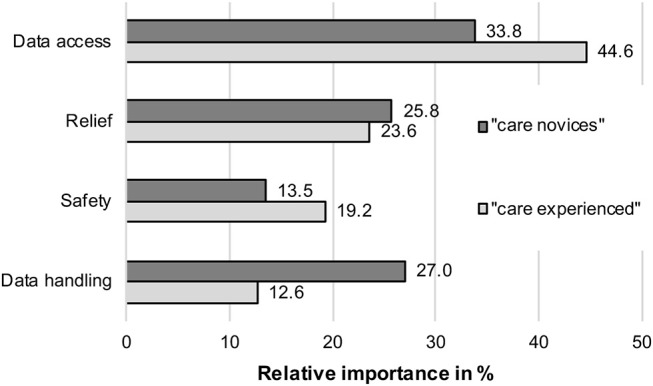
Relative importance of attributes for identified user groups.

The results of the attributes' importance and contribution to the participants' decisions are shown in Figure 4 and present the answer of RQ4.

*Privacy* (operationalized as data access) was the most important attribute for both groups, but it was even more important for the “care experienced” (44.6%) compared to the “care novices” (33.8%). The barrier *data storage* represented the second most important factor for the scenario decisions of the “care novices” (27.0%), while it was the least important decision factor for the “care experienced” (12.6%). Both groups did not differ strongly regarding the importance of the benefit *relief*; it was slightly more important for the “care novices” (25.8%) compared to the “care experienced” (23.6%). Finally, the benefit *safety* was more important for the “care experienced” (19.2%) compared to the “care novices” (13.5%).

**Figure 5:**
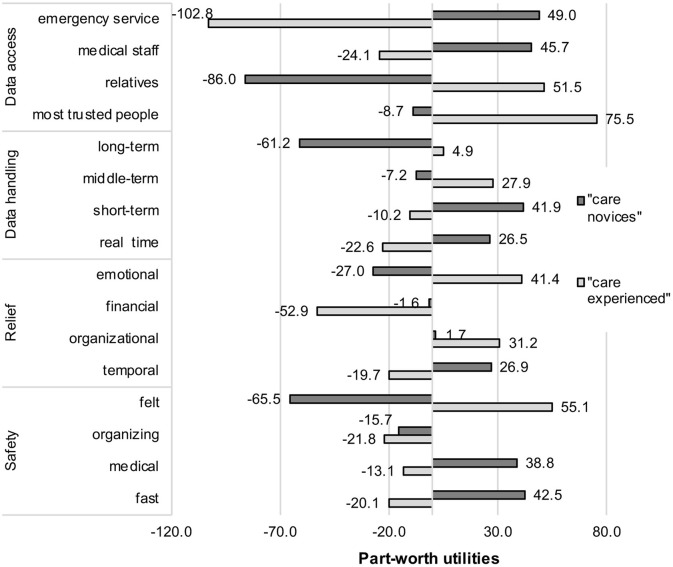
Decision patterns (part-worth utilities) regarding all attribute levels for identified user groups.

Analyzing the weight of the single attribute levels may help to understand the differences in the two groups' decision behavior. The results of the utility values of all attribute levels and for both groups are detailed in Figure 5 and represent the answer of RQ5.

It can be seen on a first sight that the trade-offs between the older care experienced participants and the younger care novices grossly differ within the single levels of each attribute.

Starting with the most important decision criterion of both groups—privacy operationalized as data access—a complete diverging decision pattern revealed. For the “care novices,” data access for emergency services (+49.0) and medical staff (+45.7) contributed positively to the scenario decisions. In contrast, for the “care experienced” group, the same attribute levels were evaluated contrariwise: Data access by medical staff (−24.1) and even stronger by emergency services (−102.8) were not desired and contributed negatively to the decisions. The evaluation of data access by relatives and a circle of most trusted people revealed again an opposite decision pattern across both groups: here, the “care experienced” perceived data access by a circle of most trusted people as best option (+75.5), while it contributed slightly negatively to the scenario decisions of the “care novices” (−8.7). When data access is enabled for relatives, this impacts positively (+51.5) the decisions of the “care experienced” to use AAL technologies at home. However, it was clearly the opposite for the “care novices” (−86.0).

For the second barrier of the AAL system's usage, data storage, once more, a diverse decision pattern showed up. A short-term storage of data (+41.9) and real-time processing of data (+26.5) received both positive utility values for the “care novices,” whereas middle-term storage (−7.2) and in particular long-term storage of data (−61.2) contributed negatively to their decisions. In contrast, middle-term storage of data (+27.9) represented the most favored option for the “care experienced,” followed by long-term storage (+4.9). Short-term storage (−10.2) as well as real-time processing (−22.6) were negatively evaluated alternatives.

Also, for the perceived benefits of AAL technology usage, the results revealed contradicting decision patterns. Starting with the benefit relief, temporal relief (+26.9) was the own aspect with a clear positive contribution to the decisions of the “care novices.” Organizational (+1.7) and financial relief (−1.6) received neutral utility values, but the fact that AAL technology would provide emotional reliefs (−27.0) contributed negatively to this group's acceptance decisions. It was exactly these emotional reliefs that were most decisive (+41.4) for the “care experienced” group, followed by organizational reliefs (+31.2), which also impacted the decisions positively. In contrast, time savings (−19.7) and, in particular, financial relief (−52.9) contributed clearly negatively to the scenario decisions of this group.

The results referring to the last beneficial attribute, increase in safety, revealed also significant and at the same time divergent evaluation patterns for both groups. For the “care novices,” fast (+42.5) and medical (+38.8) safety represented the options that militated in favor of AAL usage at home, while organizing safety (−15.7) as well as perceived felt safety (−65.5) hold negative contributions. The “care experienced” group reacted differently: The most decisive attribute level was perceived safety (+55.1) for this group, whereas all other safety facets [organizing (−21.8), fast (−20.1), medical (−13.1) safety] contributed negatively to the scenario decisions.

## Discussion

The starting point of this research was the prevailing gap between the potential of AAL technologies for the care at home, on the one hand, and the still low adoption rate on the other hand. One of the reasons that might impede a successful rollout is the missing social acceptance of the persons involved, the caregivers and the care receivers. Social acceptance, however, is a fragile good, especially in the context of care, which is characterized by sensitive and personal areas, such as age-related health and illness, and in line with patients' wishes for dignity and self-determination in spite of dependence on help (17, 57). The indisputable benefits of AAL technology—the increase in safety, the higher efficiency in the emergency case, and the possibility to live longer at home—are indeed powerful arguments for a broad rollout of assistive technologies for the care of older and health-impaired persons (17, 36). On the other hand, these benefits come along with considerable personal costs, disadvantages, and risks: They range from fears of technical failure, fears over privacy issues, and the question what data are collected and how they are used (21, 40), up to the wishes to live in dignity and intimacy at home at older age (57, 58), especially in the vulnerable context of life-end situations (59). The reasons for using AAL technology at home are very individual and they are partly emotional, partly cognitive in nature. The decision to accept AAL thus requires a careful balancing of the perceived advantages and disadvantages associated with the use of technology.

The present study provided insights into people's decision behavior regarding usage of AAL technologies indicating if perceived benefits, such as increase in safety and relief of relatives, or perceived barriers, in terms of data access and data handling, are more decisive. The findings are now discussed against the background of existing knowledge in the field of acceptance of assisting technologies. Further, the applied methodological approach is critically reflected, and limitations and suggestions for future research are given.

### Insights in Decision Behaviors in AAL Benefit–Barrier Trade-Offs

The present study empirically validated privacy to be the most decisive factor in a direct weighting of beneficial and barrier-related aspects of AAL technology usage. This finding confirms previous research in which privacy-related aspects [e.g., Peek et al. (20)] and, in particular, data access represented relevant barriers of technology usage [e.g., Offermann-van Heek and Ziefle (21), Lorenzen-Huber et al. (54), and Calero Valdez and Ziefle (40)].

The high standard deviations of the relative importance indicated a diverse decision pattern within the attribute levels. Within the privacy attribute, a user-defined circle of trusted persons was preferred to be authorized for access of AAL system-related data, while a larger circle of relatives was rejected with regard to data access. In addition, data access for emergency services was denied, while data access for the family doctor was accepted. Why should medical professionals, such as emergency services, might be rejected to have access to the medical data of a person in need of care? On the basis of the present data, we can only speculate about the reasons for this finding. Most reasonably, and this is inspired by the results of a recent study (40), users are quite reluctant to share medical data with authorities, which are not already known to them, at least in Germany. In the respective study, users reported to have a large distrust in institutions, as they have concerns about what could happen with the data supposing that the data might be used for commercial reasons without informing them as owners of the data. This fits to the findings of this study according to the fact that participants would allow data access to their family doctors and the most trusted persons. Apparently, it is, to a lesser extent, the efficient help in emergency situations that is decisive, however, even more the emotional trust and the reliability of well-known trusted persons.

When it comes to the question of data storage and handling of the data, a clear-cut picture emerges. Long-term storage—even though this would be useful for long-term treatments of patients—is clearly rejected. This finding had been also recently reported in research on AAL technology (15) and health recommender systems (40). In the latter study, the type of data was also essential: While the sharing of general health data was not seen critically by participants, when the data receiver is the family doctor or when the data contribute to a general societal benefit (used for science or the increase of knowledge for therapy and diagnosis), data on mental illnesses do not want to be shared under any circumstances. The reluctance to share data is not limited to the medical field, though. Cautious attitudes toward data sharing in general and public refusal to any long-term storage were also found in other data-sensitive domains, as e.g., in automated driving (60).

Referring to the reliefs for persons in care, AAL technology was seen positive due to its simplifications in process efficiency (organizational, temporal aspects), but also psychologically, as the emotional burden is relieved. Financial reliefs though were rejected to be relevant for the final decision to use or not use AAL technology at home. This seems odd on a first sight—as additional costs are always a serious concern in technology innovations in general (61) and digital health services in particular (11). However, when it comes to weighing of different advantages and disadvantages, financial aspects are apparently not decisive.

### Nothing Else—but Care Experience—Matters: User Profiles Regarding AAL Benefit–Barrier Trade-Offs

A major outcome of this research revealed that trade-off decisions were not homogeneous across the participants under study. Rather, we identified two conversely deciding user groups: The first group, mostly young academics, around 30 years of age, with no previous care experience, and a second group, which is, on average, 10 years older, with more life experience (being in working age or even in pension) and a profound experience in care (i.e., having a family member in need of care or having even been the caregiver for a family member). Both groups did not differ in their general attitudes toward the intention to use and the overall acceptance of AAL technology. However, their decisions and the weighing of benefits and barriers against each other seem to obey completely different mindsets and visions of technology-assisted care.

Overall, privacy-related data access was the most important decision criterion for both groups, likewise. Differences between both groups appeared in the data access options and the question who is allowed to handle the data. Care experienced persons agreed on data access for an intimate circle of confirmed persons as well as for relatives, while they were quite reluctant to allow data access for medical professionals (medical staff and emergency services). Apparently, this group wants to be in control of the family members' health condition, as they can rely on experience in caring for a family member and accept the responsibility of care. In addition, missing trust in the trustworthiness of medical services becomes evident (62, 63). Completely opposite, decisions of care novices are based on the (assumed) professional competency: Data access should thus be allowed to medical staff and emergency services, but not to family members. The question of who is allowed to handle data and to have data access is thus impacted by different heuristics across the groups. Care experienced persons' decisions rely more on emotional aspects and the close relation between the caregiver and the care receiver. Unexperienced persons, in contrast, rely on more rational reasons (competency of professionals) and entrust medical authorities with the responsibility of data access.

The question of data handling and the storage of medical data is another criterion that divides both groups. Unexperienced younger persons tend to be more critical regarding the handling of AAL system-related data. For them, only real-time processing of data and short-term storage are acceptable, while longer periods of data storage are rejected. Thus, the added value of more detailed and profound medical long-term analyses—mediated by longer storage durations—is either not perceived as beneficial or ignored. Care experience modulates also the concerns toward long-term storage and unwanted data handling. For the care experienced persons, it was the least important decision criterion among other criteria. They agreed on at least middle-term storage of data (but were also slightly positive to long-term storage) indicating that the added value of more detailed medical data analyses is acknowledged. Apparently, the well-being of the family member in need of care and technical opportunities to assist a high care quality are more important for the care experienced group than any data-storage-related issues. Interestingly, short-term storage of data and real-time processing are even declined by the care experienced group.

Another black and white decision pattern was found for the question of which relief domestic AAL technology should bring. In general, both groups agree on the benefit of assisted care at home. However, clear-cut differences show up when it comes to the relative weight of the relief options. For care novices, efficiency gains are of utmost importance, while emotional reliefs do not play a role in the final decision for or against using AAL technology at home. A possible explanation might be that this group is not really able to judge the (emotional) burdens of being a caregiver for a family member due to their missing expertise. Instead, they perceive time constraints as the most relevant aspect and wish relief most likely in this regard. A completely reverse pattern was found for care experienced persons' decisions: Emotional relief of care burdens was the most relevant, followed by organizational reliefs. Merely temporal reliefs or financial reliefs were not regarded as decision relevant. Here, it becomes obvious that the care experienced participants know what they are talking about: they are aware of the emotional burden of care, but also the responsibility of taking charge, in line with organizational challenges of integrating caring for a family member with own everyday life.

The last aspect, increase in safety, was overall more important for care experienced in contrast to the unexperienced participants. In more detail, fast and medical safety is relevant for the persons without care experience, focusing on factual safety issues (fast medical support). In contrast, the feeling of safety by AAL assistance seems to be insignificant for them as well as the safety that stems from organizational efficiency. Probably, this pattern can be reasoned with this group's limited experience in care in line with a limited empathy for the situation of being responsible for a family member in need of care. As opposed to that, it is especially the feeling of safety that is central for the willingness to use AAL technology at home for the care experienced group. All other aspects of safety do not matter for the adoption willingness of AAL technology for the care experienced.

### Limitations and Outlook

This study revealed detailed insights into decision patterns and trade-offs between benefits and barriers of using AAL technology. We identified user profiles differing in their decision patterns, which are in particular based on different levels of care experience.

Still, some limitations of the empirical approach should be considered for future research.

A first limitation regards the scenario-based approach. The care scenarios in the conjoint approach mimic real decisions by experimentally varying the relevant benefits and barriers. Still we cannot exclude that the estimated preferences might lead to higher or lower agreements/rejections in real-life contexts, representing the well-known gap between attitudes and behavior (64). In addition, our approach requested the participants to empathize with the care situation to have a close family member in need of care and to imagine that they are the family caregiver of this person taking the perspective of a caring relative. Even though the findings of the two different care experience groups were homogeneous within their decision patterns, and different across groups, the findings finally depend on the extent to which participants envisioned that they should decide for a care person who is entrusted to them. In this regard, a detailed scenario was used in order to support the participants in empathizing with the situation of having a close family member in need of care. The participants' feedback (in open comment fields) revealed that the majority of them were indeed able to empathize with the scenario very well due to the detailed, sensitive, and comprehensible description of a daily routine of the family member in need of care.

In order to get the full picture of the caring situation, future studies could examine to what extent the decision patterns change if other perspectives are considered, e.g., being the person in need of care. A further methodological limitation refers to characteristics of the choice-based conjoint analysis, in which only a limited number of attributes can be investigated at a time. The four attributes that have been examined in the present study resulted from a thorough literature review and preceding qualitative as well as quantitative studies; thus, the relevance of these attributes is undisputed. However, there still might be further benefit- or barrier-related factors (e.g., which types of data are recorded or to what extent independency of people is increased) that will impact people's decision behavior, which should be added in future research. Here, an adaptive conjoint analysis design might be useful, which allows integrating an extended number of attributes (43).

The next step after this exploratory approach regards the consideration of larger samples as well as the investigation of specific questions, e.g., the impact of different levels of prosocial behaviors and education levels. In addition, age- and aging-related attitudes and socio-economic conditions—which are prevailing in different societies and cultures—could reveal different decision patterns with regard to the benefits and the hurdles of technology-assisted care for older people and people in need of care. The fact that this study was conducted in Germany limits the outcomes to a specific perspective of one single country and its characteristics. For future studies, it would therefore be valuable and insightful to conduct cross-national comparisons integrating people's diverse cultures, their specific attitudes and perceptions of aging as well as care, and also the specific handling of aging within different countries and their respective societies.

## Conclusion

This study realized a holistic trade-off evaluation regarding perceived benefits and barriers of using an AAL system for a family member in need of care. The novelty of the findings regards the identification of distinct evaluation patterns and preferences to which extent data access, data handling, relief for caring relatives, and increased safety contribute to the final decisions to use or not use AAL. While participants showed a similar general perception and acceptance of the defined AAL system, completely different perceptions of the trade-offs between barriers (data access, data handling) and benefits (relief for caring relatives, increased safety) were identified. Life experience and expertise in caring characterized two user profiles: For care novices, the AAL system should primarily aim for fast and medical safety and time savings for caring relatives. Data should be stored not more than short term and be accessible only to medical experts and emergency services. In contrast, for care experienced participants, an AAL system should be accessible only for a defined circle of trusted people and closer family members, but not for medical experts and emergency services. Data are allowed to be stored middle term (up to 1 month), and the AAL system should primarily aim at providing a feeling of safety and an emotional relief for caring relatives.

The presented findings pave the way (1) to informing (technical) product designers and AAL providers about these different needs of potential users, (2) to supporting (professional) communication experts and policy to understand the most relevant usage motives to be addressed in public communication strategies, and (3) to creating a mutual understanding between medical professionals and care receivers about the different trust requirements for a broad adoption motivation and a sustainable rollout of AAL technologies at home.

## Data Availability

All data sets generated and used for the study are available as Supplementary Material.

## Ethics Statement

We did not seek ethical approval from the ethics committee, as our study falls in the category where no such approval is necessary in Germany. This category spans all non-invasive, non-clinical research on human subjects, where subjects are transparently informed about the purpose, aim, and risks of the studies and when these risks are reasonably low. Prior to starting the procedure, they were informed that it is of high importance to understand free opinions and attitudes on using ambient assisted living Technology and that we were very happy if they would share their opinions with us. Still, however we stressed that they are free in taking part or not and their participation was completely voluntary. The participants were not reimbursed for taking part in the study. Further, we ensured a high standard privacy protection and let the participants know that none of their answers can be referred to them as persons as data was analyzed anonymously. Demographic data were also submitted voluntarily and all participants were informed that on request their personal data would be deleted from our encrypted hard drives. After these careful explanations, the participants reported to feel well informed about the purpose and the aim of the study and their freedom to quit participation at any time. Regarding the privacy policy explanations, the participants reported to understand that high standards were applied and deliberately accepted participation. Participant privacy is a key value that our university has committed itself to uphold. From the comments in the open question fields at the end of the survey, we learnt that those participants were interested in the topic and were keen to look at the results, which we assured them to receive.

## Author Contributions

All authors listed have made a substantial, direct and intellectual contribution to the work, and approved it for publication.

### Conflict of Interest Statement

The authors declare that the research was conducted in the absence of any commercial or financial relationships that could be construed as a potential conflict of interest.
